# Response to the Letter from Garcia-Montojo and colleagues concerning our paper entitled, Quantitative analysis of human endogenous retrovirus-K transcripts in postmortem premotor cortex fails to confirm elevated expression of HERV-K RNA in amyotrophic lateral sclerosis

**DOI:** 10.1186/s40478-019-0756-9

**Published:** 2019-07-03

**Authors:** Jeremy A. Garson, Louise Usher, Ammar Al-Chalabi, Jim Huggett, Edmund F. Day, Adele L. McCormick

**Affiliations:** 1 Division of Infection and Immunity, University College London, London, UK; 2 National Transfusion Microbiology Laboratories, NHS Blood and Transplant, Colindale, London, UK; 3 School of Life Sciences, University of Westminster, London, UK; 4 Maurice Wohl Clinical Neuroscience Institute, King’s College London, London, UK; 5 Molecular and Cell Biology Team, LGC, Teddington, UK; 6 School of Biosciences and Medicine, Faculty of Health and Medical Science, University of Surrey, Guildford, UK

**Keywords:** Amyotrophic lateral sclerosis, HERV-K, Human endogenous retroviruses, RNA, RT-qPCR

In their Letter to the Editor in this issue, Garcia-Montojo, Li and Nath [5] question several technical aspects of our study recently published in Acta Neuropathologica Communications [6]. Our study, in contrast to an earlier report by Nath’s group [11], failed to find elevated expression of HERV-K RNA in postmortem premotor cortex of patients with amyotrophic lateral sclerosis (ALS). Another study by an independent group [12] also failed to confirm elevated expression of HERV-K RNA in postmortem brain tissue of ALS patients.

There are a total of five technical issues raised by Garcia-Montojo and colleagues in their Letter. Briefly, these are, i) concerns regarding our selection of controls, in particular the number of control subjects with cancer, ii) concerns regarding the number of ALS patients investigated, iii) concerns regarding the RNA integrity numbers (RINs) of the ALS patient tissues and control tissues analysed in our study, iv) concerns regarding our selection of reference genes for RT-qPCR (quantitative real-time reverse transcription polymerase chain reaction) for data normalisation, and v) our use of the RT-qPCR technique rather than alternative methods such as RNA-Seq (whole transcriptome shotgun sequencing by next-generation sequencing) for analysing the postmortem tissue samples. Each of these questions will be considered in turn:

## The selection of controls

Garcia-Montojo and colleagues point out that nearly half of our controls (11/23) had cancer, although none had cerebral metastases. However, 25% of the controls in their own study [11] also had cancer. The proportion of controls with cancer is theoretically significant because upregulation of HERV-K expression occurs in various types of tumour tissue and it is conceivable that HERV-K released into the circulation from such tumours might be detected in the cerebral tissue of controls even in the absence of cerebral metastases. We were fully aware of this theoretical possibility when we conducted our study and therefore reanalysed our data after exclusion of all controls with cancer (see Additional file 1: Figure S11 of Garson et al., [6] reproduced here). This reanalysis did not alter the key conclusion of our study that patients with ALS do not have higher levels of cerebral HERV-K RNA expression than non-ALS controls. Garcia-Montojo and colleagues also raise the question of controls with cerebral infarcts. It is true that 2 of our 23 controls had cerebral infarcts (not affecting the premotor region analysed) and that one of these, with an old infarct, had relatively high levels of HERV-K RNA expression. However, the inclusion of these two controls in our series did not significantly influence the overall data or alter the conclusion that patients with ALS do not have higher levels of cerebral HERV-K RNA expression than non-ALS controls.

## The number of ALS patients investigated

In their Letter, Garcia-Montojo et al. correctly point out that ALS is a heterogeneous syndrome with variable clinical phenotypes and the likelihood of several different pathophysiological pathways. They argue that only a subset of these different groups is associated with activation of transposable elements including HERVs and that it is therefore important that a large enough sample size is tested to capture the ALS patients in this subset. We agree with this argument but it is a weakness of their own study [11], in which HERV-K RNA expression was measured in only 11 ALS patients, more than of ours [6] in which HERV-K RNA expression was measured in 34 patients with ALS. By analysing more than three times as many ALS patients we thereby minimised the chance of missing the postulated subset with elevated HERV-K RNA expression.

## RNA integrity numbers (RINs)

RIN values are dependent upon measurements of electrophoretically separated 28S and 18S ribosomal RNAs and thus are not necessarily the most suitable measure for assessing the quality of mRNA, which is the actual target in RT-qPCR assays. A recent RT-qPCR study of 179 human brain samples [14] concluded that RIN is an unreliable measure of RNA quality from postmortem brain tissue. Other techniques which assess the quality of mRNA more directly may be therefore be preferable to use in this context [15]. Nevertheless, we elected to assess RNA quality using RIN values in our study in order to replicate as closely as possible the methods used by Li et al. [11] in their HERV-K/ALS study. Garcia-Montojo and colleagues question the RIN values of some of the samples that we analysed, stating that “some RIN values were as low as 5.” In fact only 2 of 57 samples had RIN values as low as 5 and the mean RIN value was 6.3. There is a degree of controversy about the minimum acceptable RIN but values of > 5 [4, 9] or > 3.95 [13] have been considered adequate for such studies. It has also been reported that RNA quantification using short amplicons of less than 250 bp, such as those employed in our study, is virtually independent of RNA quality as assessed by RIN values [4].

Garcia-Montojo and colleagues also point out that the average RIN number was different between our ALS patient samples and the non-ALS control samples, however the magnitude of this difference was relatively small, 6.53 and 6.05 respectively. Most importantly, we did not find any correlation between RIN values and *GAPDH*-normalised HERV-K RNA expression (see Additional file 1: Figure S4 of Garson et al., [6] reproduced here) and so do not consider this small difference in average RIN values to be of significance in interpreting the data.

## The selection of reference genes for data normalisation

The use of reference genes for RT-qPCR data normalisation is essential to control for differences in RNA quality and quantity between samples. Reference genes to be used in any particular study should be experimentally validated to ensure that they are stably expressed in both disease and control tissues as recommended by international guidelines (MIQE) [2]. Following experimental validation we selected two reference genes, *GAPDH* and *XPNPEP1* as the most stably expressed of the nine candidate genes tested. One of these (*GAPDH*) had also been used as reference gene by Li et al. [11]. It is therefore difficult to understand the criticism raised by Garcia-Montojo and colleagues against the use of a reference gene that they also selected for their own study of HERV-K RNA expression in ALS postmortem brain, and we strongly refute their assertion that it is “impossible to interpret the data”. We obtained essentially the same result, i.e. a failure to confirm elevated expression of HERV-K RNA in postmortem brain tissue of ALS patients, whether data was normalised against *GAPDH* or *XPNPEP1*. It is noteworthy that in an evaluation of 12 candidates [3] *XPNPEP1* was identified as the most suitable reference gene for use on postmortem brain tissue in various neurological diseases, including ALS.

The authors of the Letter refer to a study by Rydbirk et al. [13] to support their heterodox view that it is better to select the least stable rather than the most stable reference genes for normalisation of RT-qPCR data in this type of study. However, Rydbirk and colleagues conclude precisely the opposite of what Garcia-Montojo et al. propose, stating that “we identified the most stable reference genes to be *UBE2D2*, *CYC1*, and *RPL13* which we recommend for future RT-qPCR studies on human brain tissue …” . Furthermore, they warn against the use of unstable reference genes “in order to avoid false results”. With reference to the data reported by Rydbirk and colleagues on GSK3B RNA expression levels in the brains of Alzheimer’s and Parkinson’s disease patients, Garcia-Montojo et al. cite several papers [1, 8, 10, 16] to support their argument about the supposed benefit of using unstable reference genes to measure cerebral RNA levels by RT-qPCR in neurodegenerative diseases. However, the cited papers do not actually provide supportive evidence because they report data on protein rather than on RNA, or on peripheral blood cells rather than brain, or on mouse rather than human, or on GSK3B splice isoform ratios rather than RNA levels. Furthermore, some studies report lower levels rather than higher levels of cerebral GSK3B RNA in Alzheimer’s disease compared to normal controls [7]. All of these points undermine their arguments about the theoretical advantage of selecting unstable reference genes.

## The use of the RT-qPCR technique

The authors of the Letter also question whether the use of the RT-qPCR technique to detect transcripts in neurodegenerative diseases may not be ideal and that it might be better to use RNA-Seq, (with or without laser capture microdissection of neurons), since it controls for a possible decrease in global transcriptional activity. Alternatively, they suggest that detection of transcripts by in situ hybridisation may be considered. The reason that we elected to use the RT-qPCR technique in our study was that we were aiming to provide independent confirmation of the findings of Li et al. [11] using the same techniques that they used in their study. Thus we used the same RT-qPCR methods, including the same reverse transcription technique, the same DNase treatment method, the same PCR reagents, the same reference gene (*GAPDH*), the same thermal cycling parameters, the same 2^-ΔΔCt^ data analysis method and identical primer sets to those that they had employed. By questioning the use of RT-qPCR in our study and by asserting that “no reliable conclusion can be made” from it, Garcia-Montojo and colleagues are thereby also questioning their own study and the reliability of their own conclusions.

In summary, the authors of the Letter raise a number of questions about technical aspects of our study. We have in this response robustly defended our technical methods and conclusions but, to the extent that any of their arguments may be valid, they apply equally to their own work.

## Additional file

Additional file 1: **Figure S11.** (reproduced from Garson et al., [6]). Relative expression levels of HERV-K *gag, pol* and *env* RNA in 34 ALS and 12 non-ALS controls without cancer. a, b, and c normalised against *GAPDH*; e, f, and g normalised against *XPNPEP1.* Horizontal black lines represent geometric means. All *p* values are > 0.05, (no significant difference) varying between 0.15 and 0.77. **Figure S4.** (reproduced from Garson et al., [6]). Lack of significant correlation between RNA integrity number (RIN) and HERV-K *gag, pol* and *env* RNA relative expression levels. Data from all 34 ALS and 23 non-ALS controls are presented. a, b, c normalised against *GAPDH*; R-squared coefficient of determination values calculated in Microsoft Excel. Linear regression *p* values are shown in each graph. (DOCX 111 kb)

## Data Availability

The datasets used and/or analysed are available from corresponding authors on reasonable request.

## Abbreviations

ALS: Amyotrophic lateral sclerosis
*GAPDH*: Glyceraldehyde 3-phosphate dehydrogenase gene (reference transcript used for normalisation)
HERV-K: Human endogenous retrovirus-K (HML-2)
RIN: RNA integrity number
RNA-Seq: Whole transcriptome shotgun sequencing by next-generation sequencing
RT-qPCR: Quantitative real-time reverse transcription polymerase chain reaction
*XPNPEP1*: X-prolyl aminopeptidase 1 gene (reference transcript used for normalisation)
