# Supplementary material for: Response to the Letter from Garcia-Montojo and colleagues concerning our paper entitled, Quantitative analysis of human endogenous retrovirus-K transcripts in postmortem premotor cortex fails to confirm elevated expression of HERV-K RNA in amyotrophic lateral sclerosis
Source: Acta Neuropathol Commun. 2019 Jul 3;7:102. doi: 10.1186/s40478-019-0756-9 (PMC6607588; doi:10.1186/s40478-019-0756-9)
Supplement: Supplementary file 1 — Figure S11. (reproduced from Garson et al., [6]). Relative expression levels of HERV-K gag, pol and env RNA in 34 ALS and 12 non-ALS controls without cancer. a, b, and c normalised against GAPDH; e, f, and g normalised against XPNPEP1. Horizontal black lines represent geometric means. All p values are > 0.05, (no significant difference) varying between 0.15 and 0.77. Figure S4. (reproduced from Garson et al., [6]). Lack of significant correlation between RNA integrity number (RIN) and HERV-K gag, pol and env RNA relative expression levels. Data from all 34 ALS and 23 non-ALS controls are presented. a, b, c normalised against GAPDH; R-squared coefficient of determination values calculated in Microsoft Excel. Linear regression p values are shown in each graph. (DOCX 111 kb) [file 40478_2019_756_MOESM1_ESM.docx]

**Additional file1: Figure S11** (reproduced from Garson et al., [6]). Relative expression levels of HERV-K *gag, pol* and *env* RNA in 34 ALS and 12 non-ALS controls without cancer. **a, b,** and **c** normalised against *GAPDH*; **e, f,** and **g** normalised against *XPNPEP1.* Horizontal black lines represent geometric means. All p values are >0.05, (no significant difference) varying between 0.15 and 0.77


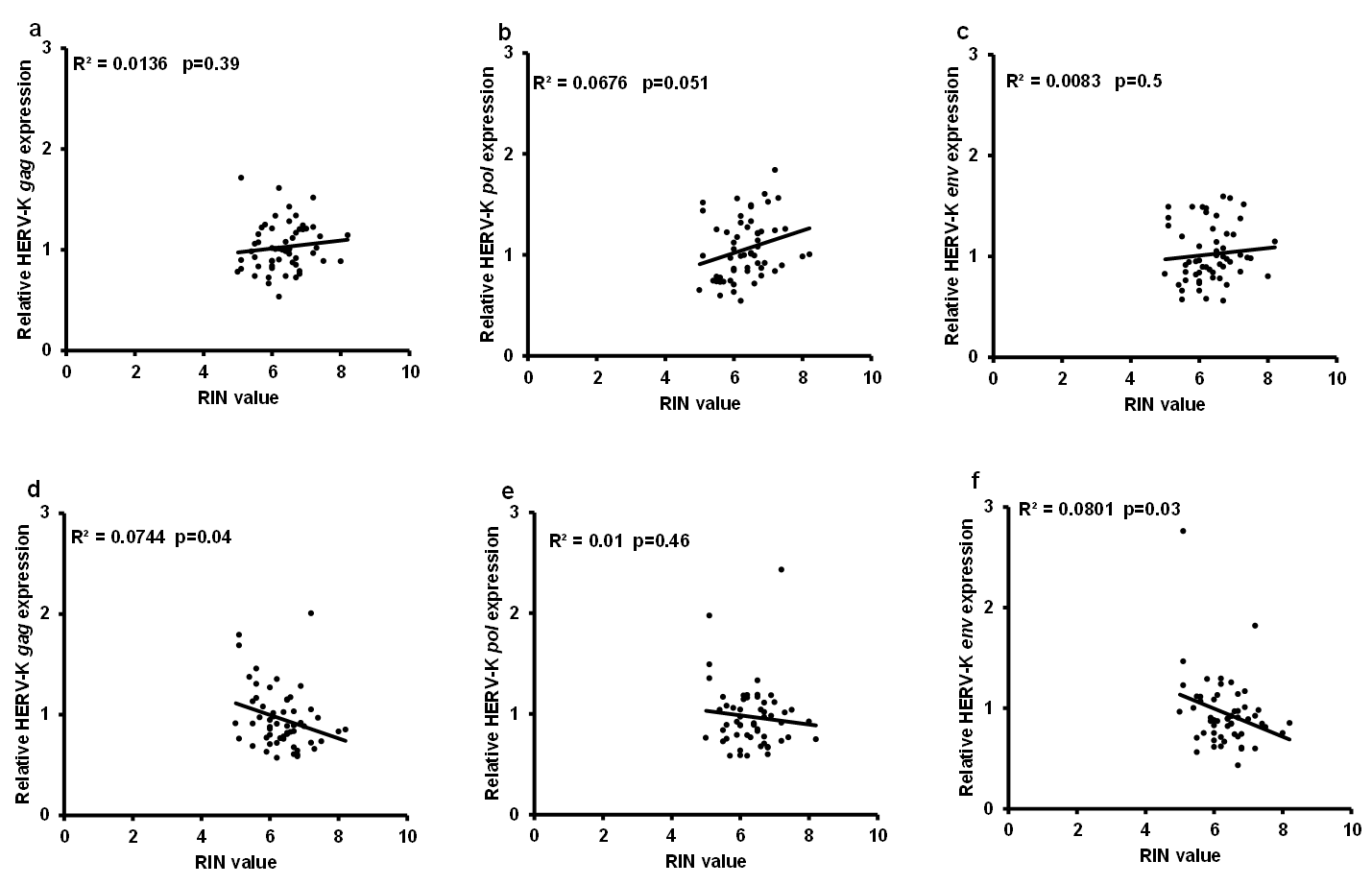


**Supplementary Fig. S4**. (reproduced from Garson et al., [6]). Lack of significant correlation between RNA integrity number (RIN) and HERV-K *gag, pol* and *env* RNA relative expression levels. Data from all 34 ALS and 23 non-ALS controls are presented. **a, b, c** normalised against *GAPDH*; R-squared coefficient of determination values calculated in Microsoft Excel. Linear regression p values are shown in each graph.
